# Human umbilical cord mesenchymal stem cells-derived exosomes deliver microRNA-375 to downregulate ENAH and thus retard esophageal squamous cell carcinoma progression

**DOI:** 10.1186/s13046-020-01631-w

**Published:** 2020-07-22

**Authors:** Zhanfeng He, Weihao Li, Tianliang Zheng, Donglei Liu, Song Zhao

**Affiliations:** Department of Thoracic Surgery, the First Affiliated Hospital of Zhengzhou University, No. 1, Jianshe East Road, Zhengzhou, 450052 Henan Province People’s Republic of China

**Keywords:** Esophageal squamous cell carcinoma, microRNA-375, Enabled homolog, Human umbilical cord mesenchymal stem cells, Exosomes

## Abstract

**Background:**

Exosomal microRNAs (miRNAs or miRs) from bone marrow-derived mesenchymal stem cells (UCMSCs) have emerged as promising therapeutic strategies for cancer treatment. The current study aimed to elucidate the underlying mechanism of human umbilical cord mesenchymal stem cells (hUCMSCs)-derived exosomal miR-375 in esophageal squamous cell carcinoma (ESCC).

**Methods:**

After determining the expression of miR-375 and its putative target enabled homolog (ENAH) in ESCC tissues and cells, we tested effects of their altered expression on ESCC proliferation, invasion, migration, and tumorsphere formation was subsequently measured. Transfected hUCMSCs-derived exosomes (hUCMSCs-exo) were isolated and co-cultured with ESCC cells to measure the effects of miR-375 delivered by hUCMSCs-exo on ESCC development. Finally, we investigated the effect of miR-375 on tumor growth in vivo.

**Results:**

The expression of miR-375 was reduced, while the expression of ENAH was elevated in ESCC. ENAH was identified as a target gene of miR-375. Elevated miR-375 or depleted ENAH expression inhibited ESCC cell proliferation, invasion, migration, tumorsphere formation, and promoted apoptosis. Moreover, miR-375 delivered by hUCMSCs-exo could suppress ESCC cell proliferation, invasion, migration, tumorsphere formation, but promoted apoptosis in vitro, as well as inhibiting tumor growth in vivo.

**Conclusions:**

Taken together, hUCMSCs-exo can deliver miR-375 to suppress ENAH expression and subsequently inhibit the initiation and progression of ESCC.

## Background

Esophageal carcinoma remains one of the most prevalent and aggressive cancers, ranking as the sixth most common cause of cancer-related deaths worldwide, and with a particularly high annual incidence rate in China [1]. Esophageal carcinoma is widely considered to have two clinical subtypes, namely esophageal adenocarcinoma and esophageal squamous cell carcinoma (ESCC) [2]. ESCC represents the more sinister of the subtypes, owing to its especially poor outcome and prognosis, which is largely due to its late diagnosis [3, 4]. Although commendable improvements have been achieved in diagnosis, surgical techniques, and post-surgical management, ESCC patients are often afflicted by ESCC recurrence [5]. Mesenchymal stromal cells (MSCs) are crucial components of the tumor microenvironment, and MSCs-derived beta-2-microglobulin is involved in the processes of epithelial mesenchymal transition, migration and tumor growth in ESCC [6]. Human umbilical cord mesenchymal stem cells (hUCMSCs) can secrete exosomes during various biological processes and hUCMSCs-derived exosomes (hUCMSCs-exos) have emerged as potential therapeutic agents [7].

As extracellular vesicles secreted by various cell types, exosomes, can serve as noninvasive biomarkers for early diagnosis of various types of cancers, and hold promise as therapeutic agents [8]. hUCMSCs-exo can alleviate inflammation and facilitate the functional recovery of mice with spinal cord injury [9]. More importantly, hUCMSCs-exo represses the progression of pancreatic ductal adenocarcinoma by delivery of exogenously loaded miR-145-5p [10]. Numerous microRNAs (miRNAs or miRs), including miR-375, have been highlighted as promising diagnostic and prognostic biomarkers for ESCC patients [11–13]. Downregulation of miR-375 is a frequent observation in ESCC, which correlates with poor prognosis, low survival rate, and tumor metastasis [14, 15]. Moreover, exosomes secreted from cells might deliver therapeutic miRs to cancer cells and neighboring cells. The exosomal miRs play a fundamental role in the development of various human diseases including cancers [16]. For example, exosomal miR-21 has been proposed as a promising target for experimental ESCC treatment [17]. In this study, our interrogation of bioinformatics data bases predicted miR-375 to target enabled homolog (ENAH). Commonly referred to as Mena, ENAH is a member of the Ena/vasodilator-stimulated phosphoprotein group and consists of actin-related proteins that play different roles in various cells [18]. Additionally, ENAH is highly expressed in gastric cancer and apparently enables its development, highlighting its potential as a prognostic marker for gastric cancer patients [19]. Based on the aforementioned exploration of literature, we hypothesized that exosomal miR-375 contributes to the development of ESCC by regulating ENAH expression. Hence, we undertook the present study to determine the underlying mechanisms of miR-375 delivered via hUCMSCs-exo in ESCC using in vitro and in vivo assays.

## Materials and methods

### Ethics statement

The study was approved by the Ethics Committee and Experimental Animal Ethics Committee of the First Affiliated Hospital of Zhengzhou University and performed in strict accordance with the *Declaration of Helsinki*. Written informed consent was obtained from all participants or their relatives prior to enrollment. Animal experiments were conducted following the recommendations in the Guide for the Care and Use of Laboratory Animals published by the US National Institutes of Health. Extensive efforts were made to minimize animal usage and discomfort during experimentation.

### Microarray analysis

ESCC-related gene expression datasets were downloaded from Gene Expression Omnibus database (https://www.ncbi.nlm.nih.gov/geo/) to screen differentially expressed genes (DEGs). A total of four gene expression datasets were obtained, comprised of two miRNA expression datasets (GSE6188 and GSE55856) and two gene expression datasets (GSE29001 and GSE20347) (Table 1). The Affymetrix R package (http://www.bioconductor.org/packages/release/bioc/html/affy.html) was used for normalization of expression data, and the limma package (http://master.bioconductor.org/packages/release/bioc/html/limma.html) was applied to screen DEGs and differentially expressed miRNAs, with |log2FC| > 1.5 and *adj.P.Val* < 0.05 as the screening criteria. Besides, an miRNA expression heatmap was constructed using pheatmap (https://cran.r-project.org/web/packages/pheatmap/index.html). The target gene of the differentially expressed miRNAs was subsequently predicted using the starBase (http://starbase.sysu.edu.cn/index.php), TargetScan (http://www.targetscan.org/vert_71/), DIANA (http://diana.imis.athena-innovation.gr/DianaTools/index.php?r=microT_CDS/index), and mirDIP (http://ophid.utoronto.ca/mirDIP/) databases. Furthermore, jvenn (http://jvenn.toulouse.inra.fr/app/example.html) was employed to screen the potential DEGs targeted by differentially expressed miRNAs, including those identified from the GSE29001 and GSE20347 datasets.

**Table 1:** Background information of ESCC-related expression datasets

Accession	Platform	Type	Organism	Sample
GSE6188	GPL4508	miRNA	*Homo sapiens*	153 ESCC cancer and 104 adjacent normal tissues
GSE55856	GPL14613	miRNA	*Homo sapiens*	108 ESCC tumor samples and matched normal samples
GSE29001	GPL571	gene	*Homo sapiens*	21 ESCC tumor and 24 normal tissue
GSE97320	GPL571	gene	*Homo sapiens*	17 ESCC tumor and matched normal tissue

Notes: ESCC, esophageal squamous cell carcinoma; miRNA, microRNA

### Tissue collection

A total of 50 ESCC patients aged 44–79 years (mean age of 62.02 ± 9.49 years) were recruited from the First Affiliated Hospital of Zhengzhou University between May 2017 and October 2018. Among them, 25 patients were diagnosed with stage I ESCC, 17 with stage II ESCC, and 8 with stage IIIa ESCC. All patients had undergone surgical resection of their respective tumors prior to initiation of chemotherapy. Both ESCC tissues and adjacent normal tissues were collected during surgery and stored at − 80 °C. All patients were pathologically diagnosed with ESCC and had not received radiotherapy or chemotherapy prior to surgery [20]. Patients who died during follow-up due to conditions unrelated to ESCC were excluded from the study.

### Cell culture

ESCC cell lines (KYSE70, ECA109, and EC9706) as well as the human normal esophageal epithelial cell line HEEC were purchased from the Shanghai Institutes for Biological Sciences, Chinese Academy of Sciences (Shanghai, China). The cells were cultured in Roswell Park Memorial Institute (RPMI)-1640 medium (Gibco, Grand Island, NY, USA) containing 10% fetal bovine serum (FBS; Gibco, Grand Island, NY, USA) at 37 °C with 5% CO_2_. The medium was changed every 2–3 days. Upon reaching 80% confluence, the cells were treated with trypsin and passaged. The cells at the logarithmic growth phase were collected for subsequent experimentation [20].

### Cell treatment

Cells at the logarithmic growth phase were detached with trypsin to prepare a single cell suspension, which was seeded into a 6-well plate. When cell confluence reached approximately 60%, miR-375 mimic, miR-375 inhibitor, plasmids overexpressing ENAH, small interfering RNA (siRNA)-ENAH as well as plasmids as negative control (NC) (NC-mimic, NC-inhibitor, ENAH-NC and siRNA-NC) were delivered into the ESCC cells and hUCMSCs alone or collectively as per the instructions of the Lipofectamine 2000 reagent (11668–019, Invitrogen, New York, California, USA). The aforementioned mimics and inhibitors were purchased from Shanghai GenePharma Co., Ltd. (Shanghai, China). siRNA-ENAH#1 (sense 5′-GGUCCUAUGAUUCAUUACATT-3′; antisense 5′-UGUAAUGAAUCAUAGGACCTT-3′), siRNAENAH#2: (sense 5′-GCGAGAAAGAAUGGAAAGATT-3′; antisense 5′- UCUUUCCAUUCUUUCUCGCTT-3′) and siRNA-NC (sense 5′-UUCUCCGAACGUGUCACGUTT-3′; antisense 5′-ACGUGACACGUUCGGAGAATT-3′) were synthesized by Guangzhou RiboBio Co., Ltd. (Guangdong, China). The eukaryotic expression plasmid containing the full length of human ENAH cDNA, pDC316-mCMV-EGFP, was purchased from Guangzhou Land Biology Company (Guangdong, China) with the empty vector applied as the ENAH-NC. A total of 100 pmol of each type of plasmid as well as 5 μL of lipofectamine 2000 were separately diluted in 250 μL Opti-minimum essential medium (MEM) and subsequently fully mixed together in the 6-well plate for transfection. Two days later, the medium was renewed with RPMI-1640 complete medium for further experiments. After 24 h of transfection, reverse transcription quantitative polymerase chain reaction (RT-qPCR) was conducted in order to determine the mRNA level. After 48 h of transfection, western blot analysis was conducted to determine the protein level.

### Dual-luciferase reporter gene assay

Firefly luciferase reporter vector carrying wild type (WT) ENAH-3’untranslated region (UTR) (PGLO-ENAH-WT) and vector expressing mutant type ENAH sequence with mutant (MUT) miR-375 binding site (PGLO-ENAH-MUT) were purchased from Guangzhou Land Biology Company (Guangdong, China). Renilla luciferase plasmid pRL-TK was regarded as the positive control. The aforementioned recombinant vectors PGLO-ENAH-WT and PGLO-ENAH-MUT were co-transfected with miR-375 mimic and NC-mimic in human embryonic kidney (HEK)-293 T cells after the cells had reached 70–80% confluence. The cells were seeded into a 12-well plate, after which the transfection process was performed using the ratio between firefly luciferase reporter vector: mimic: pRL-TK = 0.5 μg: 50 nM: 0.1 μg. After 24 h, the cells were lysed and the luciferase activity was detected using the Dual-Luciferase® Reporter Assay System (E1910, Promega, Madison, WI, USA) for firefly and renilla luciferases, whereupon the relative luciferase activity was expressed as the ratio of firefly luciferase activity to renilla luciferase activity.

### 5-ethynyl-2′-deoxyuridine (EdU) staining

ESCC cells at the logarithmic growth phase were inoculated into a 96-well plate at a density of 4 × 10^4^ cells/well in triplicates. After 24 h of culture, the cells were exposed to various treatments. After 48 h of treatment, the cells were labeled with EdU, incubated in EdU medium at 100 μL/well for 2 h. The cells were subsequently incubated with 100 μL/well cell fixative, 2 mg/mL glycine, and 100 μL/well penetrant (phosphate-buffered saline [PBS] containing 0.5% Triton X-100). The cells were stained with 1 × Apollo reaction solution and 1 × Hoechst 33342 reaction solution (100 μL/well), followed by incubation with anti-fluorescence quenching blocking solution (100 μL/well). Images were captured under a microscope for counting the number of EdU-stained cells. The cells with a red stained nucleus were regarded as positive cells. The number of positive and negative cells was determined in three randomly selected visual fields under a microscope, and the EdU staining rate (%) was calculated as the number of positive cells / (number of positive cells + number of negative cells) × 100%.

### Tumorsphere formation assay

Following a 48 h period of transfection, the ESCC cells (100 cells/well) were seeded into ultralow-adhesion 96-well plates, which had previously been coated with 10 g/L poly 2-hydroxyethylmethacrylate (HEMA) solution dissolved in absolute ethanol and air-dried for 24 h. The cells were cultured at 37 °C with 5% CO_2_ in serum-free tumor stem cell medium supplemented with Dulbecco’s modified Eagle’s medium/Ham’s F-12 medium (DMEM/F12), B27 (1:50), 20 ng/mL epidermal growth factor (EGF), basic fibroblast growth factor (bFGF), 2 mM *L*-glutamine, 0.4% bovine serum albumin (BSA), 5 μg/mL insulin, 1% knockout serum replacement and 0.8% methyl cellulose. The culture medium was changed at regular 48 h intervals. Images of tumorsphere formed after ten days were obtained under an optical microscope. The number of tumorspheres of diameter exceeding 75 μm in diameter was counted to determine the sphere formation efficiency (SFE): SFE = number of spheres/total number of cells in each well [21].

### Flow cytometry

The ESCC cells were collected for flow cytometry which was performed in accordance with the instructions of the Annexin V-fluorescein isothiocyanate/propidium iodide (FITC/PI) detection kit (MA0220, Dalian Meilun Biotech Co., Ltd. Dalian, China). Cells at a concentration of 2–5 × 10^5^ cell/mL were centrifuged at 500 g for 5 min, and resuspended in 195 μL binding buffer. The cells were subsequently incubated with 5 μL Annexin V-FITC and 10 μL PI (20 μg/mL) at room temperature in the dark, followed by flow cytometric analysis.

### Transwell assay

After 48 h of transfection, the ESCC cells were starved in serum-free medium for 24 h and then trypsinized. Next, the cells were resuspended in serum-free RPMI-1640 medium containing 10 g/L BSA, with the cell density adjusted to 3 × 10^4^ cell/mL. Cell invasion was detected by Transwell assay. Matrigel (YB356234, Shanghai Yu Bo Biotech Co., Ltd., Shanghai, China) was thawed at 4 °C overnight, and 200 μL portions were diluted with 200 μL serum-free medium. Matrigel (50 μL) was added into the apical chamber of the Transwell plates and incubated for 2–3 h until the substance had hardened. The ESCC cells were subsequently trypsinized and resuspended in RPMI-1640 medium, with the cell density adjusted to 1 × 10^5^ cell/mL. Next, 200 μL portions of cell suspension was added into the Matrigel-coated apical chamber of the Transwell plates, and 800 μL culture medium containing 20% FBS was added to the basolateral chamber. After incubation at 37 °C for 20–24 h, the Transwell plate was rinsed with 4% paraformaldehyde for 10 min. After staining of the fixed cells with 0.1% crystal violet, the chambers were allowed to settle at room temperature for 30 min. The cells on the upper surface were then carefully wiped off with a cotton ball. The cells in the chamber were photographed under an inverted microscope, and counted in a minimum of four randomly selected visual fields. The cells were incubated in non-Matrigel-coated chamber for 16 h for cell migration detection.

### hUCMSC isolation and identification

Fresh human umbilical cords were obtained from three parturient women at-term (aged 25–27 years) after Cesarean section at the First Affiliated Hospital of Zhengzhou University. hUCMSCs were collected for primary culture in accordance with the standard methods, stated below in brief [22]. The umbilical cord was rinsed with 75% ethanol and then rinsed in DMEM supplemented with 1% glutamine, 10% FBS (Thermo Fisher Scientific, Waltham, MA, USA), 100 μg/mL streptomycin and penicillin (Thermo Fisher Scientific, Waltham, MA, USA) After removal of excess blood, the umbilical cord was sliced into small pieces (3–5 mm) and incubated at 37 °C under 5% CO_2_. Upon reaching 80 to 90% confluence, the cells were trypsinized for passage. The hUCMSCs that were sub-cultured fewer than 5 times were used for subsequent experiments. The morphological cell changes were evaluated under an optical microscope (Olympus Corporation, Tokyo, Japan). The differentiation of the hUCMSCs was induced for four weeks using commercially available MSC osteogenic, chondrogenic and adipogenic differentiation kits (Cyagen, Silicon Valley, CA, USA), respectively. Next, the hUCMSCs were stained to evaluate the osteogenic, chondrogenic and adipogenic differentiation abilities based on the manufacturer’s instructions. Images were then captured using a microscope (CK40, Olympus Corporation, Tokyo, Japan).

Flow cytometry was employed to analyze the immunophenotypic characteristics of the hUCMSCs. In brief, hUCMSCs were treated with trypsin for 2–4 min, washed with calcium- and magnesium-free PBS, after which they were blocked using 10% normal goat serum to ensure low non-specific binding. The cells were then incubated with FITC-labeled antibodies against cluster of differentiation (CD)44 (338803), CD73 (344015), CD90 (328107), CD105 (323203), CD14 (367115), CD19 (392507), CD34 (343603), CD45 (368507), CD11b (301329), HLA-DR (307603) and FITC-labeled isotype control immunoglobulin G (IgG) (402006) (1:100, BioLegend, San Diego, CA, USA) for 30 min, resuspended in 10% normal goat serum and analyzed using a CyAn ADP Analyzer (Beckman Coulter, Brea, CA, USA).

### Exosome extraction and identification

After 72 h of culture in FBS-free medium, the hUCMSCs were centrifuged at 1200×g and 4 °C for 25 min to ensure removal of cell debris and dead cells, followed by filtration through a 0.2-mm filter. The resulting solution was centrifuged at 120,000×g and 4 °C for 2.5 h and centrifuged again at 120000 g at 4 °C for 2 h. The collected exosomes were resuspended in PBS and extracted for subsequent use.

To determine the characteristics of exosomes, the respective levels of the specific surface markers heat shock protein 70 (HSP70), CD63, and CD9 were measured by western blot analysis. Exosome size was determined by Zetasizer Nano ZS (Malvern Instruments, Malvern, UK). The exosomes were then allowed to settle on copper grids coated with Forvar and carbon. Copper grids were immersed in 2% phosphotungstic acid for 1 min. The morphology of exosomes was analyzed under a transmission electron microscope (TEM) (Tecnai Spirit; FEI, Hillsboro Oregon, USA) [22].

### Fluorescent labeling and transfer of exosomes

Carboxyfluorescein succinimidyl ester (CFSE) dye was diluted at a ratio of 1:1000 and subsequently mixed with 20 μg portions of exosomes secreted by hUCMSCs. The mixture was allowed to stand at 37 °C for 15 min and finally centrifuged at 100,000×g for 70 min. CFSE-traced exosomes were co-cultured with ESCC cells for 12, 24, or 48 h, whereupon the exosomes internalized by ESCC cells were visualized under a fluorescence microscope.

To determine the transfer of exosomal miR-375, hUCMSCs were transfected with Cy3-labeled miR-375 mimic (GenePharma Co., Ltd., Shanghai, China). The hUCMSCs expressing Cy3-miR-375 were then co-cultured with ESCC cells for 48 h using a 24-well Transwell chamber. Next, the ESCC cells were stained with FITC-Phalloidin (YEASEN Biotech, Shanghai, China) and the uptake of Cy3-miR-375 by ESCC cells was visualized under a fluorescence microscope.

### Co-culture of hUCMSCs-exo and ESCC cells

Exosomes were extracted from hUCMSCs that had been previously transfected with NC-mimic, miR-375 mimic, NC-inhibitor, or miR-375 inhibitor. The ESCC cells were then seeded into a 24-well plate and, upon reaching 60% confluence, hUCMSCs-exo and ESCC cells were then co-cultured for 48 h for subsequent experiments.

### RT-qPCR

Total RNA was extracted from tissues or cells based on the instructions of the TRIzol reagent (15596–018, Beijing Solarbio Science & Technology Co. Ltd., Beijing, China). The primers (Table 2) used in the study were synthesized by Takara Biotechnology Ltd. (Dalian, China). Reverse transcription was performed in accordance with the instructions of the one-step miRNA reverse transcription kits (D1801, Harbin HaiGene, Harbin, China) and complementary DNA (cDNA) reverse transcription kits (K1622, Beijing Yaanda Biotechnology Co., Ltd., Beijing, China). Fluorescence quantitative PCR was performed using the fluorescence quantitative PCR system (ViiA7, Sun Yat-sen University Daan Gene Co., Ltd., UK). Relative transcription level of target genes was calculated based on the 2^-ΔΔCt^ method with U6 and glyceraldehyde-3-phosphate dehydrogenase (GAPDH) regarded as the internal controls [23].

**Table 2:** Primer sequence for reverse transcription quantitative polymerase chain reaction

Gene	Primer sequence (5′ - 3′)
miR-375	F: AGCCGTTTGTTCGTTCGGCT
	R: GTGCAGGGTCCGAGGT
ENAH	F: TCAAGGGTAAGGGAAACTGG
	R: TGGCTCACAAGTGGTCCTCC
U6	F: CTCGCTTCGGCAGCACA
	R: AACGCTTCACGAATTTGCGT
GAPDH	F: CTCCTCCTGTTCGACAGTCAGC
	R: CCCAATACGACCAAATCCGTT

Notes: F, forward; R, reverse; miR-375, microRNA-375; ENAH, enabled homolog; GAPDH, glyceraldehyde-3-phosphate dehydrogenase

### Western blot analysis

Total proteins were extracted from tissues or cells using radio-immunoprecipitation assay lysis buffer (R0010, Beijing SolarBio Science & Technology Co. Ltd., Beijing, China). After quantification and separation via polyacrylamide gel electrophoresis, the proteins were transferred onto a polyvinylidene fluoride membrane. The membrane was blocked using 5% BSA at room temperature for 1 h and incubated at 4 °C overnight with the primary antibodies against ENAH (ab124685, 1:5000), E-cadherin (ab15148, 1:500), N-cadherin (ab18203, 1:1000), Zinc finger protein SNAI 1 (Snail; ab53519, 1:1000), B-cell lymphoma 2 (Bcl-2) (ab182858, 1:2000), B-cell lymphoma extra-large (Bcl-xl) (ab32370, 1:1000), Bcl-2-associated X protein (Bax) (ab32503, 1:5000), CD133 (ab19898, 1:1000), Nanog (ab80892, 1:1000), octamer-4 (OCT-4) (ab134218, 1:1000), CD63 (ab216130, 1:500), HSP70 (ab79852, 1:10000), CD63 (ab79559, 1:1000), Cainexin (ab22595, 1:1000) and GAPDH (ab9485, 1:2500). The membrane was incubated with horseradish peroxidase (HRP)-labeled goat anti-mouse IgG (ab205718, 1:20000) at room temperature for 1 h and then developed. All the aforementioned antibodies were purchased from Abcam, Inc. (Cambridge, UK). Protein quantification was performed using ImageJ 1.48u software (National Institutes of Health, Bethesda, Maryland, USA) with the relative protein expression was presented as a gray intensity ratio normalized to that of GAPDH.

### Hematoxylin-eosin (HE) staining

The transplanted tumor tissues were extracted, fixed with 4% paraformaldehyde for 48 h at 4 °C, and then stored in 0.02% sodium azide solution. Next, the tissues were dehydrated in an ascending series of alcohol (70, 80, and 95%, absolute ethanol, iso-butanol and finally n-butanol), paraffin-embedded and cut into sections. The paraffin sections were dewaxed with xylene for 5 min, rehydrated with gradient ethanol (100, 95, 90, 80 and 70%), and stained with hematoxylin (H8070-5 g, Beijing Solarbio Science & Technology Co., Ltd., Beijing, China) for 25–30 min. After that, the sections were differentiated with 1% hydrochloric acid ethanol for 15–30 s, and washed with water for 10–15 min until turning sky blue. Then the sections were stained for 2 min with eosin solution (PT001, Shanghai Bogoo Biological Technology Co., Ltd., Shanghai, China), followed by gradient ethanol dehydration. The sections were dewaxed twice with xylene, 1 min for each, blocked with neutral gum, dried at room temperature and labeled. Finally, the sections were observed under an optical microscope (DMM-300D, Shanghai Caikon Optical Instrument Co. Ltd., Shanghai, China) to analyze the morphological changes of transplanted tumor cells [24].

### Immunohistochemistry

The paraffin-embedded tumor tissues were deparaffinized, and dehydrated using gradient alcohol. The tissues were then washed with running water, H_2_O_2_ containing 3% methanol, distilled water, and 0.1 M PBS, followed by antigen retrieval in water bath. Goat serum blocking solution (C-0005, Shanghai Haoran Bio Technologies Co., Ltd., Shanghai, China) was added at room temperature and incubated for 20 min. The tissues were subsequently incubated with primary mouse antibody against ENAH (ab124685, 1:500, Abcam), Ki-67 (ab238020, 1:500, Abcam), E-cadherin (ab231303, 1:100, Abcam), and rabbit antibody against Bcl-2 (ab196495, 1:200, Abcam) at 4 °C overnight as well as the secondary goat anti-mouse IgG (ab6785, 1:1000, Abcam) or goat anti-rabbit IgG (ab6721, 1:1000, Abcam) at 37 °C for 20 min. After incubation with HRP-labeled streptavidin protein working solution (0343-10,000 U, Imubio™ Co., Ltd., Beijing, China) at 37 °C for 20 min, the tissues were rinsed with 0.1 M PBS and developed using diaminobenzidine (ST033, Guangzhou Weijia Technology Co., Ltd., Guangzhou, China). The tissues were then counterstained with hematoxylin (PT001, Shanghai Bogoo Biotechnology Co., Ltd., Shanghai, China) and 1% ammonia. The tissues were dehydrated using gradient alcohol and cleared using xylene. After mounting with neutral balsam, the cells were counted under a microscope in five randomly selected high-power visual fields from each section (100 cells/section). The samples were considered negatively stained when the percentage of positive cells was less than 10%, positively stained if the percentage of positive cells was between 10 and 50%, and strongly positive when the percentage of positive cells exceeded 50% [25].

### Subcutaneous xenograft tumor model in nude mice

A total of 60 specific pathogen-free female BALB/c nude mice aged five weeks and weighing 16–18 g were purchased from Shanghai SLAC Laboratory Animal Co., Ltd. (Shanghai, China). The mice were subcutaneously injected with KYSE70 cells (5 × 10^6^ cells/mL) or EC9706 cells (5 × 10^6^ cells/mL) to establish a xenograft tumor model. Next, 250 μL serum-free Opti-MEM was used to dilute 100 pmol miR-375 agomir, and 5 μL lipofectamine 2000, respectively. The aforementioned diluents were fully mixed, allowed to stand for 20 min, and then incubated with ESCC cells in a 6-well plate. After 12 h of incubation, the culture medium was renewed for an additional 48 h period of culture. Next, the exosomes were extracted from miR-375 agomir-transfected hUCMSCs and NC-agomir-transfected hUCMSCs, namely exo-miR-375 agomir or exo-NC-agomir respectively. At the 5th, 10th, 15th, 20th, and 25th day, the nude mice were administered with normal saline via tail vein injection, exo-miR-375 agomir, or exo-NC-agomir. Tumor volume (V) was determined based on the following formula: V = A (maximum diameter) × B^2^ (vertical diameter)/2 (mm^3^). The mice were euthanized at day 28 and the xenograft tumors were resected and weighed. RNA and proteins were extracted from the tumors for RT-qPCR and western blot analysis. The tumor tissues were paraffin-embedded and sliced in accordance with the standard methods for histopathological analysis.

### Statistical analysis

All data were analyzed using SPSS 21.0 software (IBM Corp. Armonk, NY, USA). Data were expressed as mean ± standard deviation. Data obeying normal distribution and homogeneity of variance between two groups were compared using unpaired *t*-test, while comparisons among multiple groups were analyzed using one-way analysis of variance (ANOVA), followed by Tukey’s test. The Kolmogorov-Smirnov method was applied to evaluate the normal distribution of data. Comparisons of cell proliferation at different time points were analyzed using repeated measures ANOVA. Differences were considered significant when *p* < 0.05.

## Results

### miR-375 is poorly expressed in ESCC

**Fig. 1:**
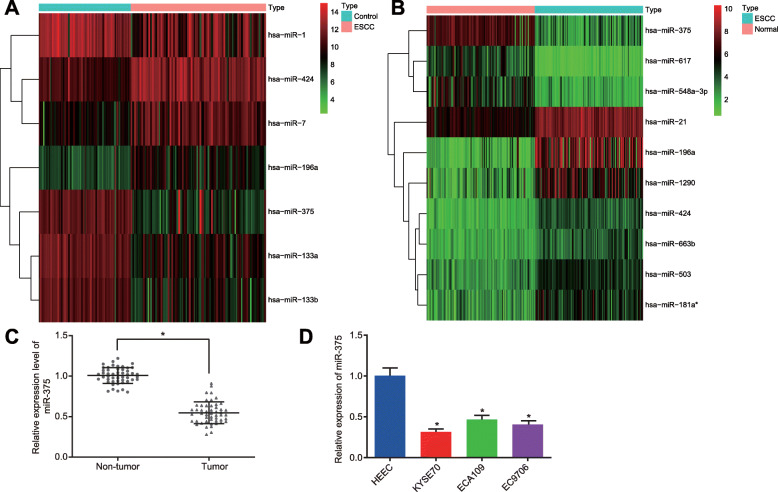
ESCC correlated to low expression of miR-375. **A**, Heatmap of the top ten miRNAs with the largest fold change in the GSE6188 dataset. **B**, Heatmap of the top ten miRNAs with the largest fold change in the GSE55856 dataset. In panel A and B, the abscissa represents sample number and the ordinate represents differentially expressed miRNAs. The upper right histogram shows a color scale, where each rectangle shows the expression of an individual sample. **C**, Expression of miR-375 was determined by RT-qPCR in ESCC tissues and adjacent normal tissues (*n* = 50), relative to U6. D, Expression of miR-375 was measured by RT-qPCR in KYSE70, ECA109, EC9706 and HEEC cells, relative to U6. * *p* < 0.05 vs. adjacent normal tissues by paired *t*-test or HEEC cells by one-way ANOVA. Data were shown as mean ± standard deviation of three technical replicates

Microarray analysis was conducted to determine the differentially expressed ESCC-related miRNAs. Hence, 7 and 27 differentially expressed miRNAs were obtained from the GSE6188 and GSE55856 datasets, respectively. Heatmaps illustrating the seven miRNAs as well as the top ten miRNAs with the largest fold change from the GSE55856 dataset were subsequently constructed (Fig. 1A, B), where intersection mapping indicated three miRNAs, namely hsa-miR-375, hsa-miR-424, and hsa-miR-196a. Among those, miR-375 was found to be an under-expressed miRNA with the largest fold change in ESCC. Thus, we focused our present study on the effects associated with miR-375 on ESCC.

ESCC tissues and adjacent normal tissues were collected from 50 ESCC patients and three ESCC cell lines (KYSE70, ECA109, and EC9706), and the HEEC cell lines were selected to determine the expression of miR-375 in ESCC using RT-qPCR. ESCC tissues had lower miR-375 expression than adjacent normal tissues (*p* < 0.05; Fig. 1C). Moreover, the miR-375 expression in HEEC cells was lower than that in HEEC cells, with the lowest expression in KYSE70 cells (*p* < 0.05; Fig. 1D). KYSE70 and EC9706 cells were therefore selected for subsequent experimentation.

### miR-375 inhibits ESCC cell malignant phenotypes but accelerates cell apoptosis

**Fig. 2:**
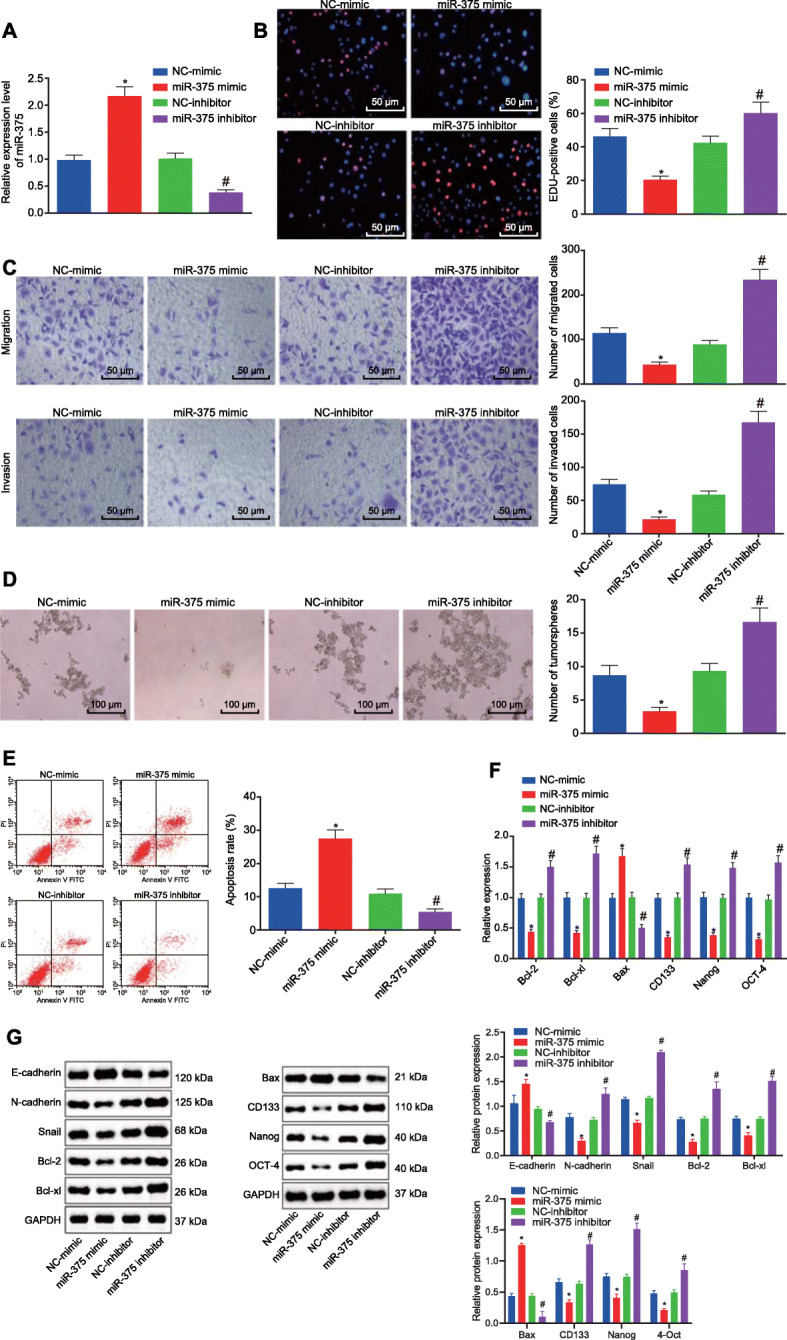
miR-375 suppressed ESCC cell proliferation, invasion, migration, and stemness while inducing apoptosis in vitro. **A**, The expression of miR-375 was determined by RT-qPCR in KYSE70 cells transfected with miR-375 mimic or inhibitor, relative to U6. **B**, Proliferation of KYSE70 cells in response to miR-375 mimic or inhibitor transfection evaluated by EdU staining (scale bar = 50 μm). **C**, Invasion and migration of KYSE70 cells in response to miR-375 mimic or inhibitor transfection evaluated by Transwell assay (scale bar = 50 μm). **D**, Tumorsphere formation of KYSE70 cells in response to miR-375 mimic or inhibitor transfection evaluated by tumorsphere formation assay (scale bar = 100 μm). **E**, Apoptosis of KYSE70 cells in response to miR-375 mimic or inhibitor transfection evaluated by flow cytometry. **F**, mRNA expression of Bcl-2, Bcl-xl, Bax, CD133, Nanog and OCT-4 was measured by RT-qPCR in KYSE70 cells, relative to GAPDH. G, Representative western blots of E-cadherin, N-cadherin, Snail, Bcl-2, Bcl-xl, Bax, CD133, Nanog and OCT-4 proteins and their quantitation in KYSE70 cells, relative to GAPDH. * *p* < 0.05 vs KYSE70 cells transfected with NC-mimic, # *p* < 0.05 vs KYSE70 cells transfected with NC-inhibitor by one-way ANOVA. Data are shown as mean ± standard deviation of three technical replicates

We performed a series of assays were performed to elucidate the effects of miR-375 on the biological characteristics of ESCC cells in vitro, using KYSE70 cells transfected with either miR-375 mimic or inhibitor. Initially, RT-qPCR was performed to determine the expression of miR-375 in KYSE70 cells, which showed the expected elevated expression of miR-375 in miR-375 mimic-transfected KYSE70 cells, and decreased expression in miR-375 inhibitor-transfected KYSE70 cells (Fig. 2A). Next, EdU staining, Transwell assay, tumorsphere formation assay and flow cytometry were performed to identify the mechanism and effects exerted by miR-375 on the biological functions of KYSE70 cells. Transfection with miR-375 mimic attenuated KYSE70 cell proliferation (Fig. 2B), invasion, migration (Fig. 2C), and tumorsphere formation (Fig. 2D) yet enhanced apoptosis (Fig. 2E), while miR-375 inhibitor exerted opposite effects on KYSE70 cellular behaviors (Fig. 2B-E).

Consistent findings were observed in EC9706 and ECA109 cells that miR-375 mimic transfection induced inhibition of cell proliferation, invasion, migration and tumorsphere formation yet promotion of apoptosis, while miR-375 inhibitor had opposite effects (Supplementary Fig. 1A-E, Supplementary Fig. 2A-E).

Furthermore, RT-qPCR and western blot analysis were employed to determine the expression of various apoptosis-related genes such as Bcl-2, Bcl-xl and Bax, stemness-related genes like CD133, Nanog and OCT-4, and epithelial-mesenchymal transition (EMT)-related proteins including E-cadherin, N-cadherin and Snail in KYSE70 cells. Bax mRNA and protein expression was elevated while that of Bcl-2, Bcl-xl and stemness-related genes was diminished in the KYSE70 cells transfected with miR-375 mimic, while opposite effects were observed in miR-375 inhibitor-transfected KYSE70 cells (Fig. 2F, G). miR-375 mimic-transfected KYSE70 cells had increased mRNA and protein expression of E-cadherin, but diminished expression of N-cadherin and Snail was detected (Fig. 2F, G). Opposite changes were identified in miR-375 inhibitor-transfected KYSE70 cells. At the same time, the consistent effects of miR-375 in relation to the mRNA and protein expression of those factors were detected in the EC9706 and ECA109 cells (Supplementary Fig. 1F, G, Supplementary Fig. 2F, G). The aforementioned results demonstrated that miR-375 suppressed the proliferation, invasion, migration and stemness of ESCC cells while stimulating ESCC cell apoptosis.

### ENAH is a target gene of miR-375 and is highly expressed in ESCC

**Fig. 3:**
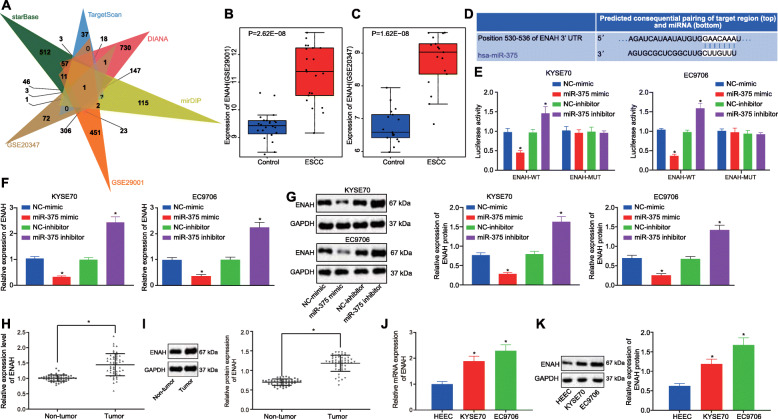
ENAH was highly expressed in ESCC and targeted by miR-375. **A**, Venn diagram showing target genes of miR-375 predicted by the starBase, TargetScan, DIANA, and mirDIP databases and DEGs in ESCC-related GSE29001 and GSE20347 microarray datasets. **B**, Expression of ENAH in ESCC and adjacent normal tissues in the GSE29001 dataset. **C**, Expression of ENAH in ESCC and adjacent normal tissues in the GSE20347 dataset. **D**, Putative miR-375 binding sites in the 3’UTR of ENAH mRNA in the TargetScan website (http://www.targetscan.org/vert_71/). **E**, Luciferase activity of ENAH-3’UTR-WT and ENAH-3’UTR-MUT in cells in the presence of miR-375 detected by dual-luciferase reporter gene assay. **F**, The mRNA expression of ENAH was determined by RT-qPCR in KYSE70 and EC9706 cells following enhancement or inhibition of miR-375, relative to GAPDH. **G**, The protein expression of ENAH was determined by western blot analysis in KYSE70 and EC9706 cells following enhancement or inhibition of miR-375, relative to GAPDH. **H**, The mRNA expression of ENAH was determined using RT-qPCR in ESCC tissues and adjacent normal tissues (n = 50), relative to GAPDH. **I**, The protein expression of ENAH was determined using western blot analysis in ESCC tissues and adjacent normal tissues (n = 50), relative to GAPDH. **J**, The mRNA expression of ENAH was determined by RT-qPCR in KYSE70, EC9706 and HEEC cells, relative to GAPDH. K, The protein expression of ENAH was determined by western blot analysis in KYSE70, EC9706 and HEEC cells, relative to GAPDH. Data in panel H and I were analyzed using paired *t*-test, in panel B and C were compared using independent sample *t*-test while in panel E-G, J and K were analyzed using one-way ANOVA. * *p* < 0.05 vs. cells transfected with NC-mimic or NC-inhibitor, adjacent normal tissues, or HEEC cells. Data are shown as mean ± standard deviation of three technical replicates

The downstream target genes of miR-375 were predicted by the starBase, TargetScan, DIANA and mirDIP databases, which revealed 821, 301, 1251, and 645 candidate genes, respectively. Moreover, microarray analysis of the GSE29001 and GSE20347 datasets screened 843 and 432 DEGs, respectively. The Venn diagram displaying the DEGs and candidate genes from four mRNA-miRNA prediction databases is depicted in Fig. 3A, where ENAH was the only intersecting gene. The expression of ENAH in ESCC tissues was higher than that in adjacent normal tissues in the GSE29001 (Fig. 3B) and GSE20347 datasets (Fig. 3C), suggesting that ENAH could be an up-regulated gene in ESCC.

The interaction between miR-375 and ENAH was further investigated. A specific binding site between miR-375 and ENAH was predicted by the TargetScan website (Fig. 3D). To verify whether ENAH was indeed a target gene of miR-375, we employed the dual-luciferase reporter gene assay. When compared with NC-mimic, miR-375 mimic transfection resulted in a reduction in luciferase activity in the ENAH-3’UTR-WT (*p* < 0.05), while no such effect was seen on ENAH-3’UTR-MUT in cells (*p* > 0.05) (Fig. 3E). In comparison to the NC-inhibitor, miR-375 inhibitor transfection elevated luciferase activity of ENAH-3’UTR-WT (*p* < 0.05) while no significant effect was identified with the ENAH-3’UTR-MUT in cells (*p* > 0.05) (Fig. 3E). To further characterize the interaction between miR-375 and ENAH, the expression of ENAH was measured after inhibition or elevation of miR-375 expression in both the KYSE70 and EC9706 cells. As illustrated in Fig. 3F, G, the expression of ENAH was reduced in cells in response to miR-375 mimic transfection, while elevated levels were detected following transfection with the miR-375 inhibitor. Taken together, these results suggested that miR-375 targeted ENAH and negatively regulated its expression in vitro.

Furthermore, ENAH mRNA (Fig. 3H) and protein (Fig. 3I) expression were elevated in the ESCC tissues compared with adjacent normal tissues (*p* < 0.05). Moreover, ENAH mRNA (Fig. 3J) and protein (Fig. 3K) expression was higher in the KYSE70 and EC9706 cells relative to that in the HEEC cells (*p* < 0.05). These results demonstrated that the expression of ENAH was elevated in ESCC.

### ENAH is involved in miR-375-mediated inhibition of ESCC progression in vitro

**Fig. 4:**
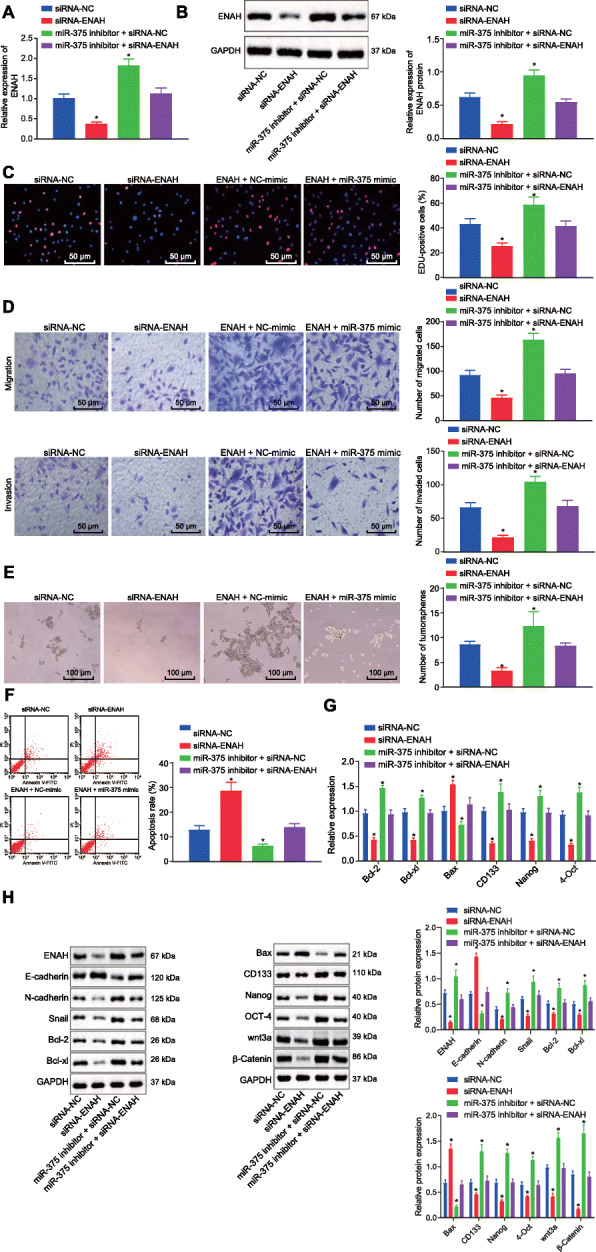
miR-375 downregulated ENAH, thus repressing proliferation, invasion, migration and stemness, and promoting apoptosis of KYSE70 cells in vitro. **A**, The mRNA expression of ENAH was determined by RT-qPCR in KYSE70 cells, relative to U6 or GAPDH. **B**, The protein expression of ENAH was determined by western blot analysis in KYSE70 cells, relative to U6 or GAPDH. **C**, Proliferation of KYSE70 cells in response to inhibition of both ENAH and miR-375 or either alone, as assessed by EdU assay (scale bar = 50 μm). **D**, Invasion and migration of KYSE70 cells in response to inhibition of both ENAH and miR-375 or either alone, as assessed by Transwell assay (scale bar = 50 μm). **E**, Tumorsphere formation of KYSE70 cells in response to inhibition of both ENAH and miR-375 or either alone, as assessed by tumorsphere formation assay (scale bar = 100 μm). **F**, Apoptosis of KYSE70 cells in response to inhibition of both ENAH and miR-375 or either alone, as assessed by flow cytometry. **G**, The mRNA expression of Bcl-2, Bcl-xl, Bax, CD133, Nanog and OCT-4 was measured by RT-qPCR in KYSE70 cells, relative to GAPDH. **H**, Representative western blots of E-cadherin, N-cadherin, Snail, Bcl-2, Bcl-xl, Bax, CD133, Nanog and OCT-4 proteins and their quantitation in KYSE70 cells, relative to GAPDH. * *p* < 0.05 vs. KYSE70 cells transfected with siRNA-NC by one-way ANOVA. Data are shown as mean ± standard deviation of three technical replicates

To determine whether ENAH participates in the development of ESCC, the expression of ENAH in KYSE70 cells was altered by ENAH overexpression plasmid or siRNA. Transfection with siRNA targeting ENAH (siRNA-ENAH) efficiently decreased mRNA and protein expression of ENAH (Fig. 4A, B), and consequently inhibited KYSE70 cell proliferation (Fig. 4C), invasion, migration (Fig. 4D), and tumorsphere formation (Fig. 4E), along with a simultaneous enhancement of apoptosis (Fig. 4F). However, elevated expression of ENAH (Supplementary Fig. 3A, B) resulted in promotion in KYSE70 cell proliferation, invasion, migration and tumorsphere formation, in addition to repressed apoptosis (Supplementary Fig. 3C-F). Meanwhile, rescue experiments were conducted to determine whether miR-375 mediates the progression of ESCC via ENAH. siRNA-ENAH attenuated the increase of mRNA and protein expression of ENAH induced by miR-375 inhibitor in cells (Fig. 4A, B), and diminished the promotion of cell proliferation, invasion, migration and tumorsphere formation and the reduction of apoptosis caused by miR-375 inhibitor (Fig. 4C-F). On the contrary, elevation of miR-375 inhibited the promotion of cell proliferation, invasion, migration and tumorsphere formation and the repression of apoptosis induced by the increase of ENAH in cells (Supplementary Fig. 3A-F). Moreover, consistent regulatory effects of miR-375 and ENAH on apoptosis, stemness and EMT were also evidenced by the changes in expression of apoptosis-related genes (Bcl-2, Bcl-xl, and Bax), stemness-related genes (CD133, Nanog, and OCT-4), and EMT-related proteins (E-cadherin, N-cadherin, and Snail) in KYSE70 cells (Fig. 4G-H, Supplementary Fig. 3G, H). Taken together, miR-375 downregulated ENAH to suppress ESCC cell proliferation, invasion, migration, stemness and promote apoptosis in vitro.

### hUCMSCs-exo can transfer miR-375 to ESCC cells

To explore the effect of hUCMSCs on ESCC cells, hUCMSCs were isolated and identified. Results of the fluorescence-activated sell Sorter (FACS) analysis revealed that the hUCMSCs were positive for CD105, CD73, CD44, and CD90, but negative for the hematopoietic markers CD34, CD45, CD14, CD19, CD11b and HLA-DR (Supplementary Fig. 4A). The hUCMSCs were fusiform-shaped, arranged in parallel and exhibited spiral growth. After oil red O staining, red lipid droplets were identified in the cytoplasm, indicating adipocyte differentiation. The alizarin red staining illustrated that mineralized nodules (osteoblasts) had formed in the middle of cell clusters. The alcian blue staining displayed that proteoglycan was stained dark blue (chondroblast) in the cartilage tissues. The above findings (Supplementary Fig. 4B) suggested that the isolated cells had multipotential differentiation abilities, which was consistent with the biological characteristics of hUCMSCs.

**Fig. 5:**
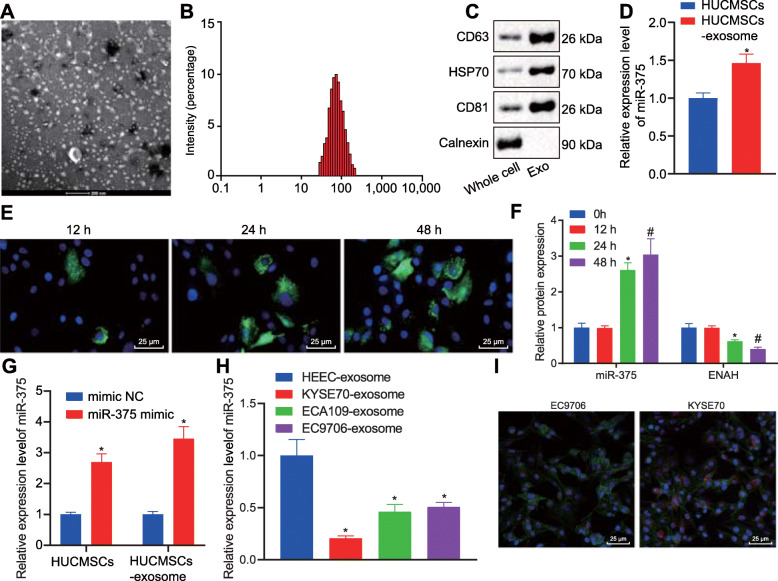
miR-375 was delivered into KYSE70 cells via hUCMSCs-exo. **A**, Morphological changes of hUCMSCs-exo observed under a TEM (scale bar = 200 nm). **B**, Diameter of hUCMSCs-exo analyzed using Zetasizer Nano ZS. **C**, Representative Western blots of hUCMSCs-exo surface marker (HSP70, CD63, and CD9) proteins and their quantitation in hUCMSCs and hUCMSCs-exo, relative to GAPDH. **D**, miR-375 expression was determined by RT-qPCR in hUCMSCs and hUCMSCs-exo, relative to U6. **E**, Uptake of hUCMSCs-exo by KYSE70 cells was observed under a fluorescence microscope (scale bar = 25 μm). **F**, Expression of miR-375 and ENAH was determined by RT-qPCR in KYSE70 cells at 12 h, 24 h, and 48 h, relative to U6. **G**, miR-375 expression was determined by RT-qPCR in hUCMSCs transfected with miR-375 mimic and the derived exosomes, relative to U6. **H**, Expression of miR-375 was examined by RT-qPCR in KYSE70-exosomes, ECA109-exosomes, EC9706-exosomes and HEEC-exosomes, relative to U6. **I**, Representative fluorescence microscopic images of miR-375 delivered into KYSE70 cells from hUCMSCs (scar bar = 25 μm). Cy3-labeled miR-375 presented in red; KYSE70 cells were stained in green; DAPI-labeled nucleus was stained in blue. Data in panel C, D, G and I were compared using *t*-test while data in panel F and H were analyzed using one-way ANOVA. * *p* < 0.05 vs. hUCMSCs, expression in KYSE70 cells at 12 h, or hUCMSCs transfected with NC-mimic. Data are shown as mean ± standard deviation of three technical replicates

Identity of exosomes isolated from hUCMSCs was confirmed under a TEM, which revealed a group of round or oval membranous vesicles (diameter 40–150 nm) with similar morphology. A membranous structure was identified at the circumference of the vesicles, with low-density cellular components located at center of the vesicles (Fig. 5A). The particle size analysis by Zetasizer Nano ZS revealed that the diameter of most of the particles ranged between 30 and 150 nm, which was consistent with the diameter of exosomes (Fig. 5B). Additionally, the presence of specific markers including HSP70, CD63, and CD9 as well as an absence of Calnexin in hUCMSCs-exo suggested the successful isolation of exosomes (Fig. 5C). Moreover, RT-qPCR determined that the expression of miR-375 was higher in purified hUCMSCs-exo than in hUCMSCs (Fig. 5D). To verify whether KYSE70 cells could uptake hUCMSCs-exo, CFSE-labeled hUCMSCs-exo were co-cultured with KYSE70 cells. Fluorescence microscope revealed that the uptake of hUCMSCs-exo by KYSE70 cells was elevated over time (Fig. 5E). Furthermore, RT-qPCR was performed to determine the expression of miR-375 and ENAH at the 12th, 24th, and 48th h in the KYSE70 cells after co-culture. As depicted in Fig. 5F, miR-375 expression in KYSE70 cells was up-regulated while ENAH expression was down-regulated over time.

Next, to reveal whether hUCMSCs-exo containing miR-375 could serve as a potential target for treating ESCC, hUCMSCs were transfected with miR-375 mimic, from which exosomes were isolated for subsequent experiments. The expression of miR-375 was increased in hUCMSCs transfected with miR-375 mimic and their derived exosomes (Fig. 5G). Additionally, the expression of miR-375 examined by RT-qPCR showed a decline in ESCC-exosomes compared to HEEC-exosomes, and the lowest miR-375 expression was found in KYSE70-exosomes (*p* < 0.05) (Fig. 5H). Meanwhile, to verify whether miR-375 was indeed delivered into KYSE70 cells via exosomes, green fluorescence-tagged KYSE70 cells were co-cultured with hUCMSCs containing Cy3-labeled miR-375. Under a fluorescence microscope, the merged images demonstrated that miR-375 had been efficiently delivered from the transfected hUCMSCs into the KYSE70 cells (Fig. 5I). Therefore, hUCMSCs-exo could deliver miR-375 into ESCC cells.

### hUCMSCs-exo deliver miR-375 to ESCC cells to suppress ESCC progression in vitro

**Fig. 6:**
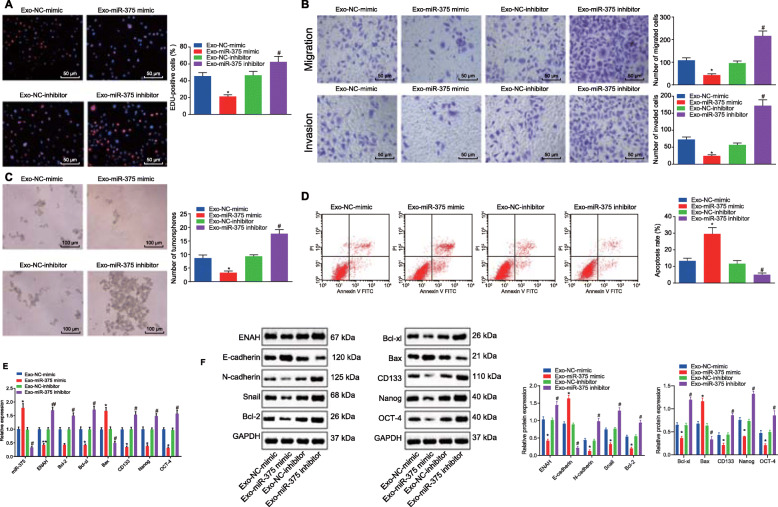
miR-375 inhibited proliferation, migration, invasion and stemness, and enhanced apoptosis of KYSE70 cells via delivery of hUCMSCs-exo in vitro. **A**, Proliferation (A) of KYSE70 cells co-cultured with exo-miR-375 mimic or exo-miR-375 inhibitor evaluated by EdU staining (scale bar = 25 μm). **B**, Invasion and migration of KYSE70 cells co-cultured with exo-miR-375 mimic or exo-miR-375 inhibitor evaluated by Transwell assay (scale bar = 50 μm). **C**, Tumorsphere formation of KYSE70 cells co-cultured with exo-miR-375 mimic or exo-miR-375 inhibitor evaluated by tumorsphere formation assay (scale bar = 100 μm). **D**, Apoptosis of KYSE70 cells co-cultured with exo-miR-375 mimic or exo-miR-375 inhibitor evaluated by flow cytometry. **E**, The miR-375 expression and mRNA expression of ENAH, Bcl-2, Bcl-xl, Bax, CD133, Nanog and OCT-4 was measured using RT-qPCR in KYSE70 cells, relative to U6 and GAPDH, respectively. **F**, Representative western blots of ENAH, E-cadherin, N-cadherin, Snail, Bcl-2, Bcl-xl, Bax, CD133, Nanog and OCT-4 proteins and their quantitation in KYSE70 cells, relative to GAPDH. * *p* < 0.05 vs. KYSE70 cells co-cultured with exo-NC-mimic; # *p* < 0.05 vs. KYSE70 cells co-cultured with exo-NC-inhibitor by one-way ANOVA. Data are shown as mean ± standard deviation of three technical replicates

To determine whether miR-375 could be delivered into ESCC cells via hUCMSCs-exo and thus inhibit the development of ESCC, exosomes derived from hUCMSCs transfected with miR-375 mimic or inhibitor (exo-miR-375 mimic or exo-miR-375 inhibitor) were co-cultured with KYSE70 and EC9706 cells, respectively. The biological functions of the cells were assessed by conducting EdU, Transwell, and tumorsphere formation assays. The exo-miR-375 mimic attenuated the proliferation, migration, invasion, and tumorsphere formation, while enhancing the apoptosis of both KYSE70 (Fig. 6A-D) and EC9706 cells (Supplementary Fig. 5A-D). However, opposite effects were evoked by the exo-miR-375 inhibitor.

Furthermore, the uptake of exo-miR-375-mimic elevated miR-375 expression and mRNA and protein expression of Bax but reduced mRNA and protein expression of ENAH, Bcl-2, Bcl-xl and expression of stemness-related mRNAs both in KYSE70 (Fig. 6E, F) and EC9706 cells (Supplementary Fig. 5E, F). The effect of exo-miR-375 inhibitor was opposite to that of exo-miR-375-mimic both in KYSE70 (Fig. 6E, F) and EC9706 cells (Supplementary Fig. 5E, F). In summary, the delivery of overexpressed miR-375 by hUCMSCs-exo inhibited proliferation, migration, invasion and stemness, in addition to enhancing the apoptosis of ESCC cells in vitro.

### miR-375 delivered by hUCMSCs-exo inhibits tumor growth in vivo

**Fig. 7:**
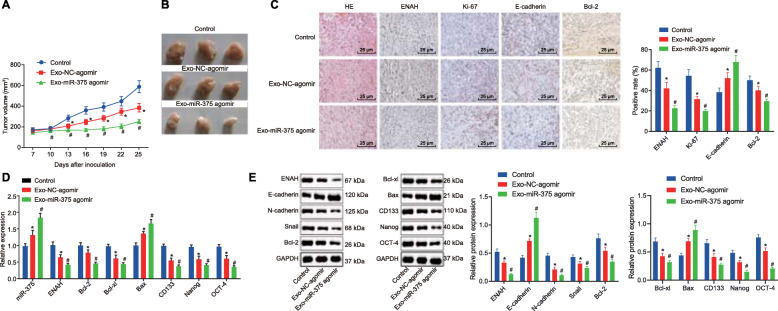
Delivery of miR-375 by HUCMSC-derived exosomes repressed tumor growth of KYSE70 cells in vivo. The tumor-bearing nude mice were injected with normal saline, KYSE70 cells treated with exo-miR-375 agomir or exo-NC-agomir. **A**, Tumor volume in nude mice. **B**, Representative images of tumors in nude mice. **C**, HE staining of mouse tumor tissues and positive expression of ENAH, Ki-67, E-cadherin and Bcl-2 proteins in tumor tissues from nude mice detected by immunohistochemistry (scale bar = 25 μm). **D**, miR-375 expression and mRNA expression of ENAH, Bcl-2, Bcl-xl, Bax, CD133, Nanog and OCT-4 was determined by RT-qPCR in tumor tissues, relative to GAPDH or U6, respectively. **E**, Representative western blots of ENAH, E-cadherin, N-cadherin, Snail, Bcl-2, Bcl-xl, Bax, CD133, Nanog and OCT-4 proteins and their quantitation in tumor tissues, relative to GAPDH. The data are shown as mean ± standard deviation. Data in panel B-E were analyzed using one-way ANOVA and data in panel A were analyzed using repeated measures ANOVA. * *p* < 0.05 vs. nude mice injected with normal saline. # *p* < 0.05 vs. nude mice injected with KYSE70 cells treated with exo-NC-agomir. *n* = 10 for mice. Following each treatment

A subcutaneous xenograft tumor model in nude mice was established via subcutaneous injection of KYSE70 cells. The nude mice were subsequently injected with normal saline or exosomes derived from hUCMSCs transfected with miR-375 agomir or NC-agomir (exo-miR-375 agomir or exo-NC-agomir) to elucidate the role of exosomal miR-375 in vivo. The mice injected with exo-miR-375 agmoir or exo-NC-agomir exhibited smaller tumors than did the mice treated with normal saline (Fig. 7A, B). The tumor volume and weight were reduced in tumor-bearing mice injected with exo-miR-375 agmoir as compared to the tumor-bearing mice injected with exo-NC-agomir (Fig. 7A, B). The tumors of mice treated with normal saline showed incomplete cell morphology, pyknotic nuclei, enlarged intercellular space, and disordered arrangement. Exo-NC-agomir treatment resulted in relatively complete cell morphology, clear staining, obviously deformed cells, vague intercellular space, and loose arrangement. However, more intact cell layers, normal size and more complete structure were observed in mouse tumor tissues following exo-miR-375 agomir treatment. Relative to normal saline treatment, exo-miR-375 agomir or exo-NC-agomir administration elevated E-cadherin expression, and decreased the expression of ENAH, Ki-67 and Bcl-2 in the tumor tissues, of which exo-miR-375 agomir led to more pronounced alterations (Fig. 7C). The aforementioned results obtained from EC9706 cells were consistent with those from KYSE70 cells (Supplementary Fig. 6A-C). Additionally, miR-375 expression and mRNA and protein expression of Bax and E-cadherin were elevated while that of ENAH, Bcl-2, Bcl-xl, N-cadherin and Snail and expression of stemness-related mRNAs were diminished in the tumor tissues of the mice injected with exo-miR-375 agomir or exo-NC-agomir relative to findings in mice injected with normal saline. The exo-miR-375 agomir provoked the more pronounced effects (Fig. 7D, E). Additionally, the aforementioned results obtained from EC9706 cells were consistent with those seen in KYSE70 cells (Supplementary Fig. 6D, E). These findings provided consistent evidence that miR-375 was successfully delivered via hUCMSCs-exo to suppress the tumor growth of ESCC in vivo.

## Discussion

Despite recent advancement in ESCC therapeutic approaches that have delivered improved patient outcomes, ESCC patients still suffer from poor five-year survival rate [26]. This highlights the importance of developing novel therapeutic targets for treating this disease. Recently, exosomal miRs exert functional effects affecting various cancer hallmarks, highlighting their potential as non-invasive therapeutic targets for cancer therapy [27]. In the current study, we found that miR-375 delivered by exosomes from hUCMSCs suppressed the initiation and progression of ESCC by downregulating ENAH.

In the first part of the study, we illustrated that miR-375 was poorly expressed in ESCC. These results were consistent with the findings of a previous study reporting reduced expression of miR-375 both in ESCC tissues and serum samples, which was correlated with a low survival rate in ESCC patients [28]. Moreover, miR-375 downregulation has been reported to be a promising prognostic biomarker for ESCC patients, and miR-375 could inhibit lymphatic vessel invasion of ESCC cells [29]. In contrast to miR-375, ENAH was found to be highly expressed in ESCC. A prior study concluded that ENAH was expressed at high levels in gastric cancer, while the upregulation of ENAH aggravated the proliferation and migration of gastric cancer in vitro and in vivo [30], which was consistent with the results of our study. Likewise, upregulated ENAH plays a tumor-promoting role in hepatocellular carcinoma [31]. These results together provide strong evidence that upregulated ENAH performs an oncogenic role in multiple types of cancers including ESCC. Additionally, ENAH has been verified as a target of miR-495 and is associated with adipose cell differentiation in MSCs [32]. A previous study demonstrated that downregulated ENAH suppressed proliferation and migration of GC cells [33]. Moreover, ENAH depletion was previously reported to suppressed migration, invasion and metastasis in hepatocellular carcinoma [34]. Likewise, ENAH deficiency inhibited cell invasion, and metastasis, thus inhibiting the progression of breast cancer [18]. Therefore, results of the current study add to the evidence that ENAH is involved in the proliferation, migration, and invasion of ESCC in addition to other types of cancers.

Previous work concurs in showing that miR-375 suppresses the proliferation, migration, and invasion of cancer cells. For example, Hu et al. showed that restoration of miR-375 expression resulted in the suppression of cell invasion and proliferation while promoting cell cycle arrest [35]. Another study showed that miR-375 overexpression suppressed migration and invasion of ESCC cells [36]. Moreover, miR-375 directly targets short hox gene 2 (SHOX2), which further inhibits the metastasis and invasion of ESCC cells. Thus, miR-375/SHOX2 was proposed as a promising future therapeutic target for ESCC treatment [20]. The aforementioned findings are consistent with present findings that miR-375 negatively targeted ENAH to suppress ESCC cell proliferation, migration, and invasion.

Another notable finding of the present study was that miR-375 delivered via exosomes could inhibit the development of ESCC. Various types of cells (including cancer cells) are capable of secreting exosomes, and exosomes derived from human MSCs mainly influence human diseases via the delivery of mRNAs, miRs, or proteins [37, 38]. For example, miR-100 delivered by exosomes secreted from bone marrow-MSCs has been reported to inhibit breast cancer angiogenesis [39]. Hence, we contend that exosomal miR-375 may suppress ESCC by inactivating the Bcl-2 signaling pathway, which has been previously identified in colon cancer [40]. Additionally, hUCMSCs have the ability to suppress the initiation and development of lung cancer and hepatocellular cancer, highlighting that hUCMSCs can exert broad tumor-suppressive effects [41]. Moreover, hUCMSCs-exo impairs the development of pancreatic ductal adenocarcinoma through delivery of exogenous miR-145-5p [10]. Hence, it is reasonable to conclude that hUCMSCs-exo indeed transfers miR-375 to ESCC cells, which could ultimately prevent ESCC progression.

## Conclusions

**Fig. 8:**
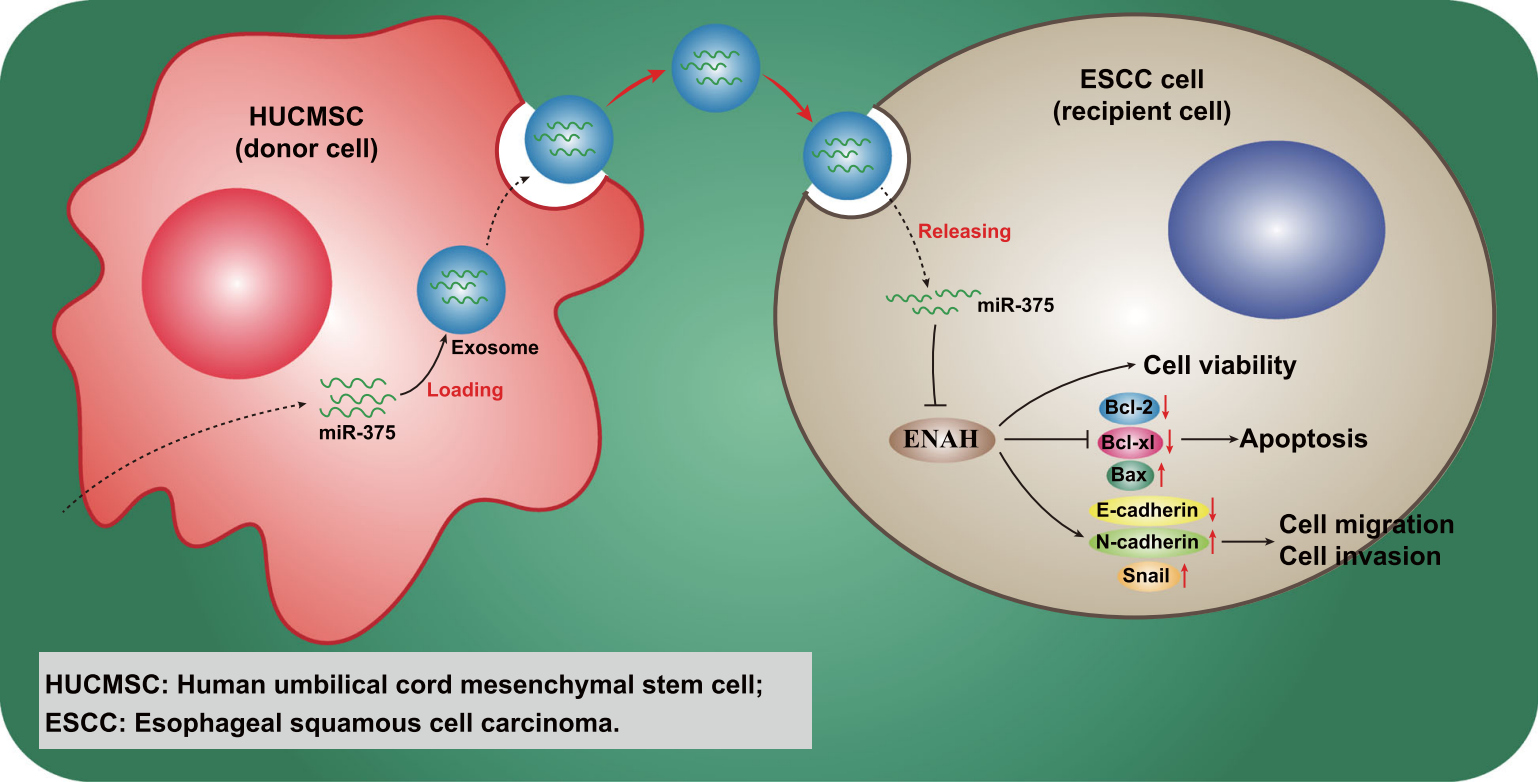
Schematic diagram showing potential molecular mechanisms by which exosomal miR-375 regulates the progression of ESCC. miR-375 is delivered into ESCC cells via hUCMSCs-exo to decrease expression of ENAH, and subsequently to increase expression of Bax and E-cadherin but to reduce expression of N-cadherin, Snail, Bcl-2 and Bcl-xl, leading to decreased proliferation, invasion and migration and enhanced apoptosis of ESCC cells

Taken together, the present study provides evidence demonstrating that hUCMSCs-exo delivering miR-375 can result in the suppression of ENAH and the initiation and progression of ESCC (Fig. 8). The central observations of our study suggest that exosomal miR-375 and ENAH may be therapeutic targets for the treatment of ESCC. However, the effects of hUCMSCs-exo with deficient miR-375 on tumor growth in nude mice remain undefined. Despite this limitation on generalizability, present findings are encouraging for the development of clinically viable targets for treating ESCC.

## Supplementary information

**Additional file 1: Supplementary Fig. 1.** miR-375 inhibited proliferation, invasion, migration and stemness and promoted apoptosis of EC9706 cells in vitro. A, Expression of miR-375 was determined by RT-qPCR in EC9706 cells transfected with miR-375 mimic or inhibitor, relative to U6. B, Proliferation of EC9706 cells in response to miR-375 mimic or inhibitor transfection evaluated by EdU staining (scale bar = 50 μm). C, Invasion and migration of EC9706 cells in response to miR-375 mimic or inhibitor transfection evaluated by Transwell assay (scale bar = 50 μm). D, Tumorsphere formation of EC9706 cells in response to miR-375 mimic or inhibitor transfection evaluated by tumorsphere formation assay (scale bar = 100 μm). E, Apoptosis of EC9706 cells in response to miR-375 mimic or inhibitor transfection evaluated by flow cytometry. F, mRNA expression of Bcl-2, Bcl-xl, Bax, CD133, Nanog and OCT-4 was measured by RT-qPCR in EC9706 cells, relative to GAPDH. G, Representative western blots of E-cadherin, N-cadherin, Snail, Bcl-2, Bcl-xl, Bax, CD133, Nanog and OCT-4 proteins and their quantitation in EC9706 cells, relative to GAPDH. * *p* < 0.05 vs. EC9706 cells transfected with NC-mimic or NC-inhibitor by one-way ANOVA. Data are shown as mean ± standard deviation of three technical replicates.

**Additional file 2: Supplementary Fig. 2.** miR-375 repressed proliferation, invasion, migration and stemness while stimulating promoting apoptosis of ECA109 cells in vitro. A, Expression of miR-375 in ECA109 cells transfected with miR-375 mimic or inhibitor determined by RT-qPCR, relative to U6. B, Proliferation of ECA109 cells in response to miR-375 mimic or inhibitor transfection evaluated by EdU staining (scale bar = 50 μm). C, Invasion and migration of ECA109 cells in response to miR-375 mimic or inhibitor transfection evaluated by Transwell assay (scale bar = 50 μm). D, Tumorsphere formation of ECA109 cells in response to miR-375 mimic or inhibitor transfection evaluated by tumorsphere formation assay (scale bar = 100 μm). E, Apoptosis of ECA109 cells in response to miR-375 mimic or inhibitor transfection evaluated by flow cytometry. F, mRNA expression of Bcl-2, Bcl-xl, Bax, CD133, Nanog and OCT-4 was measured by RT-qPCR in ECA109 cells, relative to GAPDH. G, Representative western blots of E-cadherin, N-cadherin, Snail, Bcl-2, Bcl-xl, Bax, CD133, Nanog and OCT-4 proteins and their quantitation in ECA109 cells, relative to GAPDH. * *p* < 0.05 vs. ECA109 cells transfected with NC-mimic or NC-inhibitor by one-way ANOVA. Data are shown as mean ± standard deviation of three technical replicates.

**Additional file 3: Supplementary Fig. 3.** miR-375 repressed proliferation, invasion, migration, stemness and promoted apoptosis of EC9706 cells by downregulating ENAH in vitro. A, mRNA expression of ENAH was determined by RT-qPCR in EC9706 cells, relative to GAPDH. B, protein expression of ENAH was determined by western blot analysis in EC9706 cells, relative to GAPDH. C, Proliferation of EC9706 cells in response to inhibition of both ENAH and miR-375 or either alone, as assessed by EdU assay (scale bar = 50 μm). D, Invasion and migration of EC9706 cells in response to inhibition of both ENAH and miR-375 or either alone, as assessed by Transwell assay (scale bar = 50 μm). E, Tumorsphere formation of EC9706 cells in response to inhibition of both ENAH and miR-375 or either alone, as assessed by tumorsphere formation assay (scale bar = 100 μm). F, Apoptosis of EC9706 cells in response to inhibition of both ENAH and miR-375 or either alone, as assessed by flow cytometry. G, mRNA expression of Bcl-2, Bcl-xl, Bax, CD133, Nanog and OCT-4 was determined by RT-qPCR in EC9706 cells, relative to GAPDH. H, Representative western blots of E-cadherin, N-cadherin, Snail, Bcl-2, Bcl-xl, Bax, CD133, Nanog and OCT-4 proteins and their quantitation in EC9706 cells, relative to GAPDH. * *p* < 0.05 vs. EC9706 cells transfected with ENAH-NC by one-way ANOVA. Data are shown as mean ± standard deviation of three technical replicates.

**Additional file 4: Supplementary Fig. 4.** The identification and multipotential differentiation abilities of isolated hUCMSCs. A, Expression of HUCMSC surface markers was detected by flow cytometry. B, The adipogenic osteogenic and chondrogenic differentiation abilities of hUCMSCs were assessed by Oil Red O staining, Alizarin Red staining and alcian blue staining assays, respectively, Light microscopic observation of hUCMSCs and adipogenic (left), osteogenic (middle), chondroblast (right) differentiation (scale bar = 25 μm).

**Additional file 5: Supplementary Fig. 5.** miR-375 impaired proliferation, migration, invasion and stemness, and induced apoptosis of EC9706 cells through the delivery of hUCMSCs-exo in vitro. A, Proliferation of EC9706 cells co-cultured with exo-miR-375 mimic or exo-miR-375 inhibitor evaluated by EdU staining (scale bar = 50 μm). B, Invasion and migration of EC9706 cells co-cultured with exo-miR-375 mimic or exo-miR-375 inhibitor evaluated by Transwell assay (scale bar = 50 μm). C, Tumorsphere formation of EC9706 cells co-cultured with exo-miR-375 mimic or exo-miR-375 inhibitor evaluated by tumorsphere formation assay (scale bar = 100 μm). D, Apoptosis of EC9706 cells co-cultured with exo-miR-375 mimic or exo-miR-375 inhibitor evaluated by flow cytometry. E, miR-375 expression and mRNA expression of ENAH, Bcl-2, Bcl-xl, Bax, CD133, Nanog and OCT-4 were determined using RT-qPCR in EC9706 cells, relative to U6 and GAPDH, respectively. F, Representative western blots of ENAH, E-cadherin, N-cadherin, Snail, Bcl-2, Bcl-xl, Bax, CD133, Nanog and OCT-4 proteins and their quantitation in EC9706 cells, relative to GAPDH. * *p* < 0.05 vs. EC9706 cells co-cultured with exo-NC-mimic; # *p* < 0.05 vs. EC9706 cells co-cultured with exo-NC-inhibitor by one-way ANOVA. Data are shown as mean ± standard deviation of three technical replicates.

**Additional file 6: Supplementary Fig. 6.** Delivery of miR-375 by HUCMSC-derived exosomes suppressed tumor growth of EC9706 cells in vivo. The tumor-bearing nude mice were injected with normal saline, EC9706 cells treated with exo-miR-375 agomir or exo-NC-agomir. A, The growth of ESCC xenogratf tumor in nude mice was measured every 3 days. B, Representative images of tumors in nude mice. C, HE staining of mouse tumor tissues and positive expression of ENAH, Ki-67, E-cadherin and Bcl-2 proteins in tumor tissues from nude mice detected by immunohistochemistry (scale bar = 25 μm). D, miR-375 expression and mRNA expression of ENAH, Bcl-2, Bcl-xl, Bax, CD133, Nanog and OCT-4 was determined by RT-qPCR in tumor tissues, relative to GAPDH or U6, respectively. E, Representative western blots of ENAH, E-cadherin, N-cadherin, Snail, Bcl-2, Bcl-xl, Bax, CD133, Nanog and OCT-4 proteins and their quantitation in tumor tissues, relative to GAPDH. The data are shown as mean ± standard deviation. Data in panel B-E were analyzed using one-way ANOVA and data in panel A were analyzed using repeated measures ANOVA. * *p* < 0.05 vs. nude mice injected with normal saline. # *p* < 0.05 vs. nude mice injected with EC9706 cells treated with exo-NC-agomir. *n* = 10 for mice following each treatment

## Data Availability

The datasets generated/analysed during the current study are available.

## Abbreviations

hUCMSCs: Human umbilical cord mesenchymal stem cells
ESCC: Esophageal squamous cell carcinoma
MSCs: Mesenchymal stromal cells
miRNAs or miRs: microRNAs
ENAH: Enabled homolog
DEGs: Differentially expressed genes
FBS: Fetal bovine serum
siRNA: Small interfering RNA
NC: Negative control
MEM: Minimum essential medium
RPMI: Roswell Park Memorial Institute
HEMA: 2-hydroxyethylmethacrylate
SFE: Sphere formation efficiency
RT-qPCR: Reverse transcription quantitative polymerase chain reaction
WT: Wild type
UTR: Untranslated region
MUT: Mutant
HEK: Human embryonic kidney
EdU: 5-ethynyl-2′-deoxyuridine
PBS: Osphate-buffered saline
DMEM/F12: Dulbecco’s modified Eagle’s medium/Ham’s F-12 mediuM
BSA: Bovine serum albumin
EGF: Ermal growth factor
FITC/PI: Fluorescein isothiocyanate/propidium iodide
HSP70: Heat shock protein 70
TEM: Transmission electron microscope
CFSE: Carboxyfluorescein succinimidyl ester
cDNA: Complementary DNA
GAPDH: Glyceraldehyde-3-phosphate dehydrogenase
Bcl-2: B-cell lymphoma 2
Bcl-xl: B-cell lymphoma extra-large
Bax: Bcl-2-associated X protein
OCT-4: Octamer-4
HRP: Horseradish peroxidase
IgG: Immunoglobulin G
HE: Hematoxylin-eosin
ANOVA: Analysis of variance
EMT: Epithelial-mesenchymal transition
FACS: Fluorescence-activated sell Sorter
SHOX2: Short hox gene 2
